# The experience of community health workers training in Iran: a qualitative study

**DOI:** 10.1186/1472-6963-12-291

**Published:** 2012-08-31

**Authors:** Sara Javanparast, Fran Baum, Ronald Labonte, David Sanders, Zohreh Rajabi, Gholamreza Heidari

**Affiliations:** 1 South Australian Community Health Research Unit (SACHRU), Flinders University, Adelaide, Australia; 2 Southgate Institute for Health, Society and Equity, Flinders University, Bedford Park, Adelaide, Australia; 3 Institute of Population Health, The University of Ottawa, Ottawa, Canada; 4 School of Public Health, University of the Western Cape, Cape Town, South Africa; 5 Behvarz Training Centre, Kashan University of Medical Sciences, Kashan, Iran; 6 Dean’s Office, Boushehr University of Medical Science, Boushehr, Iran; 7 Room 2.09, level 2, Health Sciences Building, Flinders University, Bedford Park, Flinders, SA 5042, Australia

**Keywords:** Community health workers, Training, Primary health care

## Abstract

**Background:**

The role of Community Health Workers (CHWs) in improving access to basic healthcare services, and mobilising community actions on health is broadly recognised. The Primary Health Care (PHC) approach, identified in the Alma Ata conference in 1978, stressed the role of CHWs in addressing community health needs. Training of CHWs is one of the key aspects that generally seeks to develop new knowledge and skills related to specific tasks and to increase CHWs’ capacity to communicate with and serve local people. This study aimed to analyse the CHW training process in Iran and how different components of training have impacted on CHW performance and satisfaction.

**Methods:**

Data were collected from both primary and secondary sources. Training policies were reviewed using available policy documents, training materials and other relevant documents at national and provincial levels. Documentary analysis was supplemented by individual interviews with ninety-one Iranian CHWs from 18 provinces representing a broad range of age, work experience and educational levels, both male and female.

**Results:**

Recognition of the CHW program and their training in the national health planning and financing facilitates the implementation and sustainability of the program. The existence of specialised training centres managed by district health network provides an appropriate training environment that delivers comprehensive training and increases CHWs’ knowledge, skills and motivation to serve local communities. Changes in training content over time reflect an increasing number of programs integrated into PHC, complicating the work expected of CHWs. In-service training courses need to address better local needs.

**Conclusion:**

Although CHW programs vary by country and context, the CHW training program in Iran offers transferable lessons for countries intending to improve training as one of the key elements in their CHW program.

## Introduction

The role of Community Health Workers (CHWs) in improving access to basic healthcare services, and mobilising community actions on health is broadly recognised. The Primary Health Care (PHC) approach, identified in the Alma Ata conference in 1978 [1], stressed the role of CHWs in addressing community health needs. Delegation of tasks to community level health workers has more recently been considered as a response to the global shortage in human resources for health and a key strategy to improve access to quality health services [2].

Literature on CHWs principally focuses on a few key aspects, including their tasks and activities, selection and recruitment process, training, remuneration (volunteer versus paid workers), and support system. CHW training, in turn, generally seeks to develop new knowledge and skills related to specific tasks and to increase CHWs’ capacity to communicate with and serve local people [3]. Improving access to basic training has also been found to be an important element of improving CHW retention [4].

There are many approaches to CHW training, from short term courses to long term certificate programs. CHWs in Brazil receive an 8 week residential course that includes curative, preventive and promotive components, 4 weeks of fieldwork followed by on-going training sessions [5]. In contrast, in Thailand CHWs are trained for 7 days on the concepts of PHC, disease prevention and basic curative tasks followed by on-the-job training for 15 days [5]. Training content varies significantly by the educational qualifications of CHWs and the required competencies for their roles and responsibilities, ranging from use of nationally-produced training modules to locally tailored curricula, residential courses or mobile training teams. Various forms of distance education have also been trialed to provide CHW training, although limited access to technology and low ITC literacy have been barriers to distance training in many developing countries [6].

This paper describes the training of CHWs in Iran and how the process and quality of existing training are perceived by the Iranian CHWs themselves. Our findings add to the understanding of the factors that increase the effectiveness of CHW training.

## Background

A national CHW program has existed in Iran since 1979 although there is evidence of a few pilot programs that go back decades [7,8]. Iranian CHW, called *behvarz* in the Farsi language, is a full time employee of the health system, is selected from her/his own community and works in the village Health House, the most peripheral health delivery facility in the rural areas of Iran. National, provincial and district health systems are responsible for planning and implementing CHW-related policies and programs. According to the most recent statistics, in 2007 there were about 17,000 Health Houses in Iran, staffed by almost 31,000 male and female CHWs providing services to most of Iran's 65,000 villages with an estimated population of 28 millions [9].

From the inception of the CHW program in Iran, training has been a key component that has undergone regular review based on changes in health patterns, *behvarz* qualifications and new demands in their roles. In this paper we focus on *behvarz* training including training centres and trainers, training content, duration and facilities, and how these elements are perceived by *behvarz* to affect their performance and work satisfaction.

## Methods

Information about *behvarz* training was gathered from both primary and secondary sources. Secondary data were collected through analysis of policy documents, unpublished reports, national and provincial operational plans, and training materials/modules. Documentary analysis was supplemented by individual interviews with ninety-one *behvarz* from 18 provinces. This sample was drawn from the national database of the Iranian ministry of health. Study participants were purposively recruited from differing socioeconomic and geographic areas and represented a broad range of age (from 25 to 54 years old), work experience (from 2 months to 30 years), educational levels (primary school to university student), and both male and female *behvarz*. Three Iranian-based research assistants travelled to the 18 provinces (6 each) and conducted the interviews. In each province, a number of eligible participants were approached by the interviewers and assistance from the Division of Behvarz at Provincial and District Health Centres. Those who expressed interest in participating contacted the research assistant by phone to organize an interview session. Primary data provided an in-depth understanding of the strengths and weaknesses of the training process from the viewpoint of *behvarz*.

The interviews were conducted between October 2009 and February 2010 and took place at the village Health Houses or District Health Centres depending on the preferences of participants. Interviews were recorded with the consent of participants and transcribed by the three research assistants. All audio tapes were checked against the transcribed text by the first author (SJ). Interview data were coded to comparable categories. The key themes and illustrative quotes were translated into English. This paper presents participants’ perceptions and experiences with the training process and courses. Ethics approval was granted by the Iranian ministry of health and Flinders University ethics committee.

## Results

The findings presented here are part of a larger study that investigated the contribution of *behvarz* to the implementation of comprehensive primary health care in Iran. The results of the *behvarz*-related policy review, and an analysis of their roles and functions, are presented elsewhere [10,11]. With regard to *behvarz* training, findings clustered under three critical issues: a) training centres, facilities and trainers; b) training content and duration; and c) training quality and outcome.

### Training centres, facilities and trainers

#### Pre-service training

**Figure 1:**
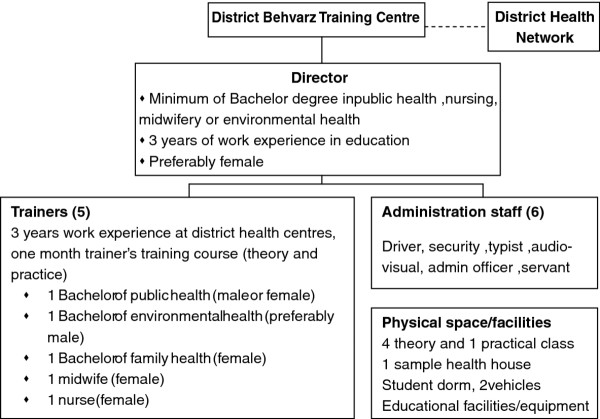
Organisation chart of District Behvarz Training Centre in Iran.

After recruitment, successful applicants undergo pre-service training. *Behvarz* pre-service training in Iran is hosted by a specialised centre called District Behvarz Training Centre (DBTC) that provides 2 year residential training for students. There are now 224 DBTCs throughout the country [9] that are linked to and supervised by the district health networks. Each centre, located at district level, consists of 1 director, 5 trainers, and administration staff. Figure 1 shows the organisational chart of DBTC in Iran. 

The DBTC is responsible for a range of activities: a) *behvarz* recruitment by having a representative in the district recruitment committee, identifying rural areas with a shortage of *behvarz*, advertisement and communication with rural councils and families to nominate the best candidates; b) administering the entrance exam and interviews with the candidates; c) planning and implementation of training courses, and administering the final exams; d) providing supervision and support during the course of training; and e) providing a safe and secure environment for students, and the arrangement of cultural or entertaining events. The DBTC also collaborates with other centres including maternity facilities; AIDS, thalassemia and family planning consultation services; and district hospitals as part of *behvarz* clinical placement program.

*Behvarz* trainers are full-time employees of the health system. Tertiary qualification in a public health-related field and a minimum of 3 years work experience in a local primary health care system are pre-requisites for DBTC trainers. Additionally, they are required to undergo one month special training as teachers, which includes placement in the DBTC, rural and district health centres. The trainers’ roles are explicitly defined and include collaboration in *behvarz* recruitment, training and day to day supervision and assessment of *behvarz* students during training.

In each centre, a range of 7–15 students are trained each year; the program restricts the size of training group to allow close supervision and contact. *Behvarz* students are provided training allowance, free training, accommodation, transport and meals during their training course. Cultural, religious and entertaining events are included to build relationship with the trainers, improve social and interpersonal communication skills and provide an enjoyable training environment for students. The cost of *behvarz* training is included in the annual budget of the national and provincial health systems.

#### In-service training

In-service training aims to update *behvarz* with new policies and programs, reinforce initial training, and ensure they are practicing skills learned. In-service training is provided at regular intervals, varying from monthly to twice a year, and offered in the form of workshops, monthly meetings and refresher courses.

Usually due to the distance between DBTC and the village Health Houses where *behvarz* work, as well as the limited number of trainers who coordinate training activities within the DBTC, in-service training is mainly carried out by GPs or other allied health workers in Rural Health Centres. Rural Health Centre is the next level of referral within the PHC network, covers 5–6 village Health Houses, and is staffed by a GP and a few allied health workers. These centres are responsible for *behvarz* supervision and also provide in-service training as part of their regular meetings with *behvarz*[12].

Although in-service training is believed to be crucial in updating their knowledge and skills, a number of participants in our study compared it unfavourably with pre-service training. They complained about its quality and timing, the infrequency of courses, inadequately qualified trainers who are unfamiliar with the *behvarz* working environment, the lack of practical sessions and of physical space and training facilities. A typical comment was:

"In-service courses are usually run by doctors or other health professionals from rural and district health centres. The quality is not comparable with what we had in the DBTC. We had closer relationship with our trainers and they knew our strengths and weak points. (female, 41 yrs)"

"We have in-service training at least once a month but usually they talk about several items in one session which is very tiring. (male, 34 yrs)"

#### Training duration and content

*Behvarz* student receives two years of residential pre-service training. The relatively long period of initial training reflects the variety and complexity of work roles that the Iranian CHWs are expected to perform, ranging from case detection and disease management in different age groups to disease prevention, health promotion and community development [11]. Training of *behvarz* is incorporated into the national PHC plan, and in turn is supported by financial and human resources for such training.

The training program was originally divided into three blocks of 6.5, 9 and 7.5 months respectively. This was changed in 2001 to a two term program consisting of “theoretical” and “practical” knowledge and skills, and “clinical placements” in Health Houses and Rural Health Centres. The inclusion of different training approaches, particularly clinical placements that allow students to gain experience in the work environment, was frequently reported by the participants to have a positive impact on their clinical and communication skills and confidence.

"The best part of our training was the time that I spent in the health house under the supervision of an experienced behvarz. I’ve learnt a lot from her which increased my skills and confidence. (female, 32 yrs)"

"The theory and practical classes were useful in increasing our knowledge but I worked for a few months in the health house in my own village as a student. This was the unforgettable part of our training course. (male, 38 yrs)"

Review of the training policies and materials demonstrated that training content has been updated regularly through a series of national and provincial meetings and workshops to ensure that it covers an appropriate range of health topics and addresses the changing needs of the target population.

**Figure 2:**
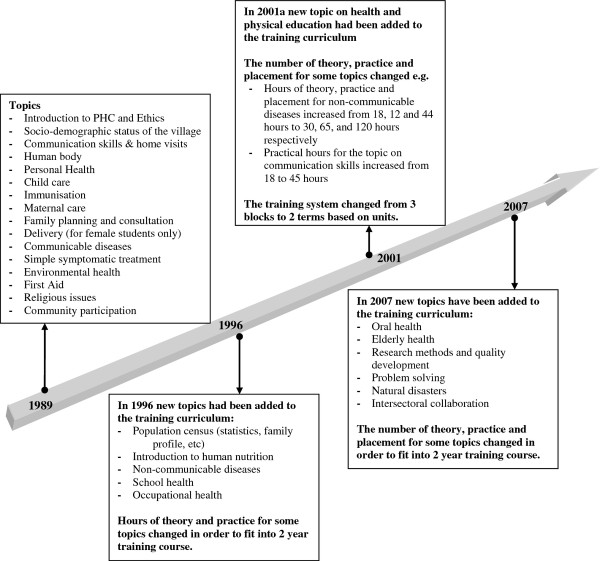
*** Behvarz* training topics between 1989 and 2007.**

As shown in Figure 2 the original training of *behvarz* had a focus on maternal and child health, communicable diseases and environmental health. The inclusion of new topics including non-communicable diseases, school health, oral health, elderly health, research methods and statistics, intersectoral collaboration, and natural disasters over time demonstrates a responsive training system to meet the changing needs of *behvarz* and rural community over time. Table 1 compares length and total hours of *behvarz* training between 1978 and 2007.

**Table 1:** **Length and total hours of *behvarz* training in Iran **

**Length of training (months)**	**Total hours of training**	**Year**
22	2996	1989
23	3672	1996
19.5	2719	2001
23	3341	2007

The importance of ongoing revision in training content was reported by a few participants:

"I started my course 30 years ago when I only had year 3 primary certificate. The training materials were very simple to match my literacy level. But now all behvarz students have at least high school certificate and so their training needs to be at a higher level and quality. (female, 48 yrs)"

The current minimum required qualification for *behvarz* candidates is high school certificate. However, due to an increased rate of rural literacy a number of candidates have university degrees in public health related fields. This group of candidates has to go through similar recruitment process including the entrance exam and interview but the duration of pre-service training decreases to 6–8 months. The training focus of this group of *behvarz* students is on improving their practical and communication skills and to fill in their knowledge gaps in areas that are not directly related to their university courses.

In some areas, in addition to the two year compulsory training, complementary training has been provided to meet the local needs. Midwifery training for female *behvarz*, called *behvarz-mama* in the Farsi language, is an example. The midwifery training is designed for rural areas that do not have access to a maternal facility and specialist midwifery services. The program includes 5 weeks training, incorporating 46 hours of theory and 252 hours placement in DBTC and public hospitals or other maternal health facilities under the supervision of *behvarz* trainers. Each student has to undertake 5 deliveries with assistance and 10 deliveries and postnatal care independently, pass the theory exam and complete the clinical placement in order to be awarded a certificate that allows graduates to conduct home deliveries in their villages. As a result of this initiative the number of deliveries by traditional birth attendants in one province decreased from 16.6% to 3% between 2006 and 2008, with a concomitant increase in home deliveries by *behvarz-mamas* from 6.4% to 12% [13].

#### Quality and outcome of training

The CHW training is mainly evaluated by an assessment of their satisfaction, and competency in delivering the tasks that are allocated to them [3]. *Behvarz* knowledge and skills are assessed through theory and practical exams during and at the conclusion of the pre-service training as pre-requisites to their certification. Similarly, pre- and post-tests are used in the in-service courses to assess knowledge improvement and gaps. However, the review of training policies and plans in Iran found little documentation of mechanisms to evaluate the quality and impact of training courses [10].

This study investigated the quality of training courses (pre- and in-service) from the viewpoint of *behvarz* and how the training courses have impacted on their daily performance. The majority of participants believed that the pre-service training was comprehensive and included relevant topics that had a huge impact on their capacity to provide healthcare services, and to build their confidence and skills in communicating with rural people.

"The quality of our training was excellent. All the knowledge and skill I gained was from these classes. It [training] doubled my interest on working as a behvarz. Unforgettable indeed… (male, 44 yrs)"

The friendly environment of the training centres, the nature of trainer-trainee relationships, and highly qualified trainers were particularly noted by most participants as features that made the training courses an exciting period of their life and career and had positive impact on the learning process and motivation.

"Behvarz training centre was the most appropriate place for our training, we were as a whole family working together. It was not just about teaching and learning a few topics, it was more about learning life skills, so motivating… (male, 22 yrs)"

"Before attending the training courses I was too shy to even talk to people but when I finished the course, I became a different person. Now I can easily communicate with people, attend meetings and work with other sectors in my village. The combination of theory classes and practice was really good… (female, 39 yrs)"

From the viewpoint of some participants, training courses broadened their understanding of *behvarz* roles and functions.

"When I started the course, I thought we are supposed to work like a nurse but after that [training courses] I realised that health education, disease prevention and promotion is our main job. (male, 34 yrs)"

Clinical placement in the Health Houses under the direct supervision of the trainers was stated crucial in gaining work experience, building relationship with local community, and collaborating with other local organisations.

"During our placement we got to know people and our future work environment. Direct supervision by trainers helped us build up our confidence and to be trusted by the people. (female, 29 yrs)"

Interviews with a broad range of *behvarz*, however, identified a number of problems related to the pre-service training. Centrally produced materials, booklets, and step-by-step guidelines was perceived didactic by some participants that constraints adult participatory learning, and problem solving capabilities of the students. This perspective was particularly prominent among younger participants with higher educational qualification.

"There are too many step-by-step guidelines and instructions that we have to follow, we are not given a chance to search, to think and to analyse things. (male, 28 yrs)"

Given the socioeconomic, geographic and climate diversity in Iran that creates differences in health needs, a number of participants stressed on the lack of formal mechanism to adapt training materials to local conditions.

"We are all trained standard booklets but different regions have different priorities. Some topics that we were taught are not very useful in our daily practice. Well, we spent many hours on an infectious disease which is not common in our region at all. I haven’t seen even one case during the last few years. (male, 40 yrs)"

## Discussion

In common with other healthcare providers the competency of CHWs encompasses knowledge, skills, abilities and traits which are largely gained through pre-service education, in-service training, and work experience [14]. There are also a number of other factors including personal motivation, working conditions, and ongoing support that affect their long term effectiveness and performance [15-17]. This study assessed the key elements of the CHW training program in Iran, how these elements are perceived by CHWs, and the lessons that may be applicable to similar settings.

Firstly, CHW training is integrated into the Iranian primary health care system and is consequently recognised in the national health planning regulations and financing. Nationally-coordinated set of CHW policies with adequate financial and resource support for training, standardised training modules, periodic reviews and certification for CHW training facilitates the implementation and sustainability of the program [18]. Effective coordination and planning at different levels of health system and the insertion of CHWs program in the wider health system have been considered crucial in successful training of CHWs [5,19].

Secondly, the existence of specialised training centres, managed by the district health system, was perceived effective in delivering comprehensive training for CHWs. It appears from this study that the general climate of the training organisation, trainer-trainee relationship and the norms of the training groups are as important as the acquisition of knowledge in motivating and stimulating CHWs to learn and apply certain skills. Residential pre-service training at district level was perceived influential in building the relationship between behvarz students and trainers as well as providing the opportunity for closer supervision and student assessment. However, this may not applicable in all settings as the ideal location for CHW training varies in different countries depending on funding, support system, facilities and the broader socio-cultural context [3,20,21].

Thirdly, CHW training content and its changes over time in Iran reflects: a) overall increasing trend in literacy rate among rural population including CHWs themselves that requires a comprehensive training to meet the educational needs of *behvarz* students; b) increasing number of health programs integrated into the Iranian PHC, and as a result complex work being expected of CHWs. The training content also demonstrates a focus on disease prevention, health promotion and health education as the principles of PHC approach, and a shift towards a social determinants approach in training as a response to the current international and national health agendas. This is visible in training content by the inclusion of topics on the health system and rural community, communication skills, social sectors in rural areas and intersectoral collaboration, social determinants of health and well-being, and social aspects of the behvarz-client relationship.

Fourthly, it appears from this study that the higher flexibility in the content of in-service training provides an opportunity to fill in the training gaps and to address local and community needs. Better management of in-service courses and greater involvement of DBTCs in facilitating such courses may improve the quality of in-service training and *behvarz* satisfaction.

Finally, this study did not set out to measure the effect of training on *behvarz* performance and community health outcomes, which is methodologically complex. However qualitative methods, such as those used in this study, can provide insights into the contextual factors and aspects of health worker training that work well in specific settings [16]. Other studies suggest that the *behvarz* program has positively affected community health gains and narrowing rural–urban gaps in health [22,23]. Although it is hard to directly attribute the health achievements to the quality of training courses, it is not illogical to suggest that the *behvarz* training program has contributed to these positive health gains.

## Conclusion

Training of CHWs is an integral part of Iranian primary health system and considerable attention has been made to make sure that the training content, duration and approaches matches with the educational qualification of CHW students as well as community health needs. However, the length of the training course and its content raise questions about how far the Iranian Behvarz can be compared with community health workers in other developing countries where the community health workers typically receive very little initial training and are more akin to lay workers than professionals.

The experience of CHWs training in Iran provides valuable lessons for countries that established CHW models and intend to provide rigorous training as one of the key program elements, although the total number of 91 interviews may not represent the full perspective of the more than 31,000 behvarz now working in rural areas of Iran. Furthermore, its applicability in terms of depth, length and training approaches vary considerably based on the context, nature of CHWs program, qualification of CHWs, expected roles and the finance system. The high level political support given in Iran to comprehensive primary health care, including the Behvarz program, is demonstration of how such support can lead to a strong health sector which contributes to improving population health outcomes and reducing urban – rural health inequities.

## Competing interests

The authors declare that they have no competing interests.

## Authors’ contributions

SJ designed the study, supervised its implementation, analysed the data, and led the preparation of the article. FB provided mentoring in the implementation of the study and assisted in the preparation of the article. RL and DS provided advice in the implementation of the study and assisted in the preparation of the article. ZR assisted in interviewing with community health workers and preparation of the article. GH coordinated research activities and assisted in the preparation of the article. All authors read and approved the final version of the article.

## Pre-publication history

The pre-publication history for this paper can be accessed here:

http://www.biomedcentral.com/1472-6963/12/291/prepub
